# Comparative Study on the Efficiency of Mercury Removal From Wastewater Using Bacterial Cellulose Membranes and Their Oxidized Analogue

**DOI:** 10.3389/fbioe.2022.815892

**Published:** 2022-03-14

**Authors:** D. Suárez-Avendaño, E. Martínez-Correa, A. Cañas-Gutierrez, M. Castro-Riascos, R. Zuluaga-Gallego, P. Gañán-Rojo, M. Peresin, M. Pereira, C. Castro-Herazo

**Affiliations:** ^1^ School of Engineering, Universidad Pontificia Bolivariana (Pontificia Bolivariana University), Medellín, Antioquia; ^2^ Tourist and Agroindustrial Technological Complex of the West Antioquia, Servicio Nacional de Aprendizaje (National Training Service), Santafé de Antioquia, Antioquia; ^3^ Forest Products Development Center, School of Forestry and Wildlife Sciences, Auburn, AL, United States; ^4^ Departamento de Ingeniería Química, Universidad de Concepción, Concepción, Chile

**Keywords:** bacterial nanocellulose, *Komagataeibacter medellinensis*, TEMPO oxidation, mercury removal, wastewater

## Abstract

A comparative study was conducted on the efficiency of mercury removal using bacterial nanocellulose (BNC) membranes obtained from the fermentation of the microorganism *Komagataeibacter medellinensis*, in contrast with its oxidized analog obtained by modifying the bacterial nanocellulose membranes *via* oxidation with 2,2,6,6-Tetramethylpiperidine-1-oxyl. Both types of membranes (modified and unmodified) were characterized to identify variations in the Physico-chemical parameters after modification. FTIR spectra confirmed the chemical modification of cellulose in all reaction conditions by the presence of a new characteristic band at ∼1730 cm^−1^, corresponding to the new carboxylic groups produced by the oxidative process, and the decline of the band at ∼1,650 cm^−1^, corresponding to the hydroxyl groups of the C6 carbon. While the XRD profiles indicated that the percentage of BNC crystallinity decreased and the SEM images showed that the nanoribbon network was interrupted as the amount of oxidizing agent increased. The kinetics of mercury removal from both types of membrane was evaluated by calculating the concentration of mercury at different times and establishing a mathematical model to describe the kinetics of this process. The modified membranes improved significantly the adsorption process of the metal ion and it was found that the modification that results in the greatest adsorption efficiency was BNC-m 7.5 with a value of 92.97%. The results obtained suggest that the modification of the bacterial nanocellulose membranes by oxidation transcendentally improves the mercury removal capacity, outlining the modified membranes as an excellent material for mercury removal in wastewater.

## Introduction

The population growth leads to an increase in the industrial production of goods, many of these use raw materials obtained from mining activities. Mining has a significant impact on the growth of environmental problems in terrestrial and aquatic ecosystems. Wastewater contaminated with heavy metals is released directly or indirectly into the environment each day, which is a huge problem due to the persistence of these pollutants in nature (Fu and Wang 2011; Yu et al., 2013a; Hokkanen 2014). In Colombia, there is evidence of environmental pollution in water sources and the air by mercury, and there have been reports of its presence in birds and humans in areas of high influence mining, such as Bajo Cauca and the north of Antioquia (Olivero et al., 1995; Olivero V and Jhonson R 2002; Olivero-Verbel et al., 2006). It is estimated that the amount of mercury released into the atmosphere as a result of anthropogenic activities is on average 4,000 tons per year (Jackson, T. A. 1997). The contamination resulting from human economic activities may arise from waste discharge, direct emissions into the atmosphere from the burning of fossil fuels, incineration of solid waste and smelting of metals such as copper and zinc (Olivero V and Jhonson R 2002).

Mercury contamination in tropical areas, particularly in Brazil, Colombia, Ecuador, and Bolivia, is mostly a product of gold mining processes. This process consists in mixing the crushed rock that contains the precious metal with elemental mercury (Hg0) to form the amalgam and subsequently removing the excess of mercury by hand. This process results in large amounts of mercury being disposed of in rivers, lakes, swamps, among others. The gold-mercury amalgam is burned in an open field to free the gold, which causes the direct release of mercury vapors into the atmosphere. Mercury and all its compounds are toxic, volatile, persistent, and quickly dispersed in the environment, in particular the species of methylmercurates, considered to be the most toxic forms of this metal (Olivero V and Jhonson R 2002).

In recent years, priority has been given to the implementation of methodologies to monitor mercury levels in the environment because it is a confirmed fact that this metal can cause adverse effects to human health and the environment (Wang et al., 2004; Wei et al., 2014). Thus, environmental procedures should be applied to remove mercury in contaminated water. Control in the generated sources and contaminated sediment remediation, or their combination, are usually used for cleaning up mercury-contaminated sites (Wang et al., 2004). In the remediation, different methods have been utilized for eliminating heavy metals from wastewater like physical, chemical, biological or combination of those (Zhou et al., 2004). Conventionally physicochemical techniques are used, that include precipitation, filtration and electrochemical recovery, and also membrane separation, excavation and deposition of residues in landfills. However, these techniques are expensive and not very efficient (Giovanella et al., 2017).

In recent years, adsorption has been used as an effective strategy for the separation of contaminants due to adsorbent content is rehabilitated, reused and recycled and it is considered as the best technique, but high cost of sorbents restricts its use (Varsha et al., 2022). The use of adsorbates of natural origin is promising, since in addition to being friendly to the environment and human health, they are usually easy to obtain and low-cost. Among these adsorbates of natural origin, natural fibers have been widely studied, especially those that are residual products of agricultural activities since they become a useful alternative when analyzing the cost-benefit aspect. In these investigations, sugar cane bagasse (Gupta at al. 2001; Kadirvelu at al. 2001; Mohan et al., 2015), rice husks (Ajmal at al. 2003; Chuah at al. 2005), coffee husks (Azouaou et al., 2010), banana husks (Anwar et al., 2010), cocoa husks (Meunier et al., 2003) have been used, among other. Clays and biopolymers have also been used for this purpose, among which chitosan (Wan Ngah et al., 2011) and cellulose (Yu et al., 2013a; Dwivedi et al., 2014; Jain 2014; Carpenter et al., 2015; Hokkanen et al., 2015; Wei et al., 2015; Zhu et al., 2015; Daneshfozoun et al., 2017) stand out, for which promising results have been obtained.

Cellulose is the most abundant biopolymer on earth and can be produced by plants, animals and microorganisms, or it can also be synthesized enzymatically (cell-free system) (Ullah t al. 2015, Kim et al., 2019).

The efficiency in the removal of heavy metals such as Cu(II), Ni(II), Pb(II), Co(II) and Zn(II), among others, have been reported using this biopolymer, since it is a raw material affordable and cheap. However, it has a low adsorption capacity, as well as variable physical stability. To overcome these drawbacks, chemical modifications have been made to achieve adequate structural durability, as well as more efficient metal adsorption (O'Connell et al., 2008; Hokkanen 2014).

Most of these reports use nanocellulose of plant origin, but to think of a treatment with this nanocellulose, it is required to isolate it by chemical-mechanical processes and then make its modification. Unlike plant cellulose, the bacterial nanocellulose (BNC) is obtained with greater purity and crystallinity (Ross et al., 1991), since it is free of lignin and hemicellulose, which makes its isolation and purification simpler, without the need for energetically or chemically intensive processes (Keshk 2014). In addition, this material is very appealing for removing pollutants due to its high surface area to selectively adsorb metals from wastewater (Ul-Islam et al., 2021; Shoukat et al., 2019), due to its nanometric structure, with a ribbon width of 20–70 nm (Castro et al., 2011), and due to its high reactivity given by the presence of hydroxyl groups on its surface, which allow it to be chemically modified to interact with different pollutants depending on its nature (Voisin et al., 2017). Other important properties of BNC have been identified such as its biodegradability, high mechanical properties, and low density (Madivoli et al., 2016).

Given that water treatment processes with biomembranes have elicited general interest in recent years, due to the need to implement efficient, low-cost and environmentally friendly pollutant removal mechanisms (Lu et al., 2014; Cacicedo et al., 2016; Jin et al., 2017), the present study assessed the efficiency of removing mercury using BNC membranes obtained from the fermentation of the microorganism *Komagataeibacter medellinensis*, in contrast with membranes that have undergone a chemical modification process (BNC-m) through oxidation, using 2,2,6,6-Tetramethylpiperidine-1-oxyl (TEMPO) as a mediator, since the carboxylate groups on the surface of the cellulose, generated during this modification, have been shown to maximize the adsorption capacity of metal ions (Voisin et al., 2017). In addition, the effect of the concentration of sodium hypochlorite (NaClO) as an oxidizing agent in the BNC modification on the mercury adsorption capacity was evaluated and correlated with the morphological changes that the material underwent after being oxidized.

## Materials and Methods

### Chemicals

Chemicals. Citric acid, glucose, peptone, yeast extract, monobasic sodium phosphate (NaH2PO4), monobasic potassium phosphate (KH2PO4), magnesium sulfate (MgSO4), potassium hydroxide (KOH), 2,2,6,6-Tetramethylpiperidine-1-oxyl (TEMPO), sodium bromide (NaBr), sodium hypochlorite (NaClO), sodium hydroxide (NaOH), hydrochloric acid (HCl) and mercury (II) nitrate (Hg(NO3)2). All reagents were purchased from Sigma-Aldrich. All the solutions were prepared using Milli-Q ultrapure water.

### Preparation of the pre-inoculum

The culture medium was prepared, containing: glucose 0.54% (w/v), peptone 0.5% (w/v), yeast extract 0.5% (w/v), monobasic sodium phosphate 0.267% (w/v), monobasic potassium phosphate 0.059% (w/v) and magnesium sulphate 0.025% (w/v), the pH was adjusted to 5 with citric acid and then sterilized at 120°C for 20 min. Subsequently, the medium was inoculated with *Komagateibacter medellinensis*. Then, the inoculated medium was incubated at 28 °C for 32 h on an orbital shaker at 100 rpm to allow the biomass growth. After the incubation process, the solution was separated into conical tubes and centrifuged at 9,000 rpm and 8 °C for 20 min. The supernatant was discarded and the pellet was washed with sterile water with subsequent repetition of the centrifugation process. The pellet obtained was redispersed in a sterile saline solution.

### Production of BNC

The culture medium was prepared, containing: glucose 2% (w/v), peptone 0.5% (w/v), yeast extract 0.5% (w/v) and disodium phosphate 0.267% (w/v), the pH was adjusted to 3.6 with citric acid and then sterilized at 120°C for 20 min. Subsequently, the medium was inoculated with the pre-inoculum 10% (v/v). Then, the inoculated medium was incubated at 28°C for 15 days on static conditions to allow the fermentation process. After the incubation process, the obtained membranes were purificated with KOH 5% (v/v) and then rinsed with fresh water until pH 7.

### Oxidation process

Bacterial nanocellulose membranes were modified (BNC-m) using the method proposed by Saito et al. with some modifications (Isogai et al., 2011). BNC membrane was suspended in a solution containing: TEMPO (0.107 mmol/mmol NaClO) and NaBr (0.133 mmol/mmol NaClO) in deionized water under shaking. Subsequently, the oxidizing agent (NaClO) was added dropwise. The amount of NaClO in each case was varied using five different proportions and the amounts of the catalysts TEMPO and NaBr were established, while preserving the stoichiometric ratios set out above (Table 1).

**TABLE 1:** Modifications were carried out with their respective variables and denominations.

Amount of NaClO (mmol)	Amount of TEMPO (mmol)	Amount of NaBr (mmol)	Denomination
2.5	0.268	0.333	BNC-m 2.5
5.0	0.535	0.665	BNC-m 5
7.5	0.803	0.998	BNC-m 7.5
10.0	1.070	1.330	BNC-m 10
15.0	1.605	1.995	BNC-m 15

The pH of the solution was maintained at about 10.0–10.5 through the addition of 0.5 M aqueous NaOH. The reaction was adjudged to have been completed when no more of the NaOH was needed to maintain the pH, the mixture was quenched by adding ethanol and the membranes were washed with plenty of water. Finally, the modified membranes were suspended in water and HCl 0.5 M was added dropwise to reach a pH = 7 to neutralize the carboxylate groups obtained after modification.

### Characterization of the Membranes

The characterization of the modified (BNC-m) and unmodified membranes (BNC) was carried out to determine if the modification was effective and to evaluate if the oxidation treatments affected the structural properties of the BNC membranes. The details for each instrumental technique are presented below.

#### Fourier Transformed Infrared Spectroscopy- Attenuated Total Reflectance (FTIR-ATR)

To determine if the chemical modification occurred, the FTIR spectra for BNC and BNC-m were obtained using a Thermo Scientific Nicolet 6700 infrared spectrophotometer equipped with an Attenuated Total Reflection (ATR) module between 4,000 and 500 cm^−1^ with a resolution of 4 cm^−1^ and an accumulation of 256 scans. The evaluation was conducted three times on each side (front and rear) for each of the membranes.

#### X-ray diffraction

Diffractograms were obtained using a Rigaku^®^ diffractometer equipped with a copper source to verify the crystallinity for each of the membranes studied. The diffraction patterns were obtained for 5°<2θ < 50° with a step of 0.02°.

#### Scanning Electron Microscopy (SEM)

Nanocellulose membranes morphology was assessed to determine changes in the nanocellulose network before and after the oxidation process. The membranes were cut, freeze-dried, coated with gold/palladium using an ion sputter coater, and observed using a JEOL JSM 6490 LV microscope under high vacuum with Secondary Electron Detector (SEI) for high-resolution images at 20 kV accelerating voltage. The analyses were performed using magnifications of 5000X and 10000X.

After the adsorption process, the modified and unmodified membranes were analyzed by energy-dispersive x-ray spectroscopy using a JEOL JSM 6490 LV electron microscope with an Energy Dispersive Detector (EDS) for elemental analysis and mapping.

### Mercury Removal Profiles

To determine the mercury removal profiles in the BNC and BNC-m membranes, an aqueous solution of ∼4 ppm mercury was used, prepared from a reference standard of mercury nitrate. The pH of the solution was adjusted to six by adding NaOH 0.5 M.

The removal trials were carried out by placing each BNC or BNC-m membrane in 200 ml of mercury solution (pH∼6), with constant orbital shaking (100 rpm). The progress of the removal was monitored by extracting 1 ml of solution at different times over 24 h.

The amount of mercury associated with each aliquot was measured by direct mercury analysis using a Milestone^®^ DMA-80 direct mercury analyzer.

### Adsorption Kinetics

To carry out the kinetic study, first, the adsorption capacity of the BNC and BNC-m membranes was calculated in each time t (qt) and equilibrium when t→∞ (qe) using Equations 1, 2, where C0 is the initial concentration of the solution (mg/L), Ct is the concentration of mercury in time t (mg/L), Ce is the concentration of mercury in equilibrium (mg/L), V is the total volume of the mercury solution (L) and m is the weight of the membrane (g) (Yu et al., 2013b). The adsorption efficiency EAd was also calculated using Equation 3 (Renuka R and K 2013).

$$q_{t}=\frac{\left(C_{0}-C_{t}\right)V}{m}$$ (1)

$$q_{e}=\frac{\left(C_{0}-C_{e}\right)V}{m}$$ (2)

$$E_{Ad}=\frac{C_{0}-C_{e}}{C_{0}}x100$$ (3)

To conduct the kinetic study of the adsorption process, the three kinetic models generally considered for these purposes were used: the Lagergren or *pseudo-first-order* model, the *Ho* or *pseudo-second-order* model, and *the intraparticle diffusion* model (Aksu 2002; Vadivelan and Vasanth Kumar 2005; Zheng et al., 2010a; Vasco et al., 2014). Table 2 presents the equations related to each proposed kinetic model.

**TABLE 2:** Kinetic model equations.

Kinetic model	Equation	Linear form
*Pseudo-first order*	$\frac{dq_{t}}{dt}=K_{1}\left(q_{e}-q_{t}\right)\left(4\right)$	$log\left(q_{e}-q_{t}\right)=logq_{e}-\frac{K_{1}}{2.303}t\left(5\right)$
*Pseudo-second order*	$\frac{dq_{t}}{dt}=K_{2}{\left(q_{e}-q_{t}\right)}^{2}\left(6\right)$	$\frac{t}{q_{t}}=\frac{1}{h}+\frac{1}{q_{e}}t\left(7\right)$
*Intraparticle diffusion*	*—*	$q_{t}=K_{int}t^{1/2}\left(8\right)$

$K_{1}$ (min^−1^) and K_2_ (g/(mg/min)) are the pseudo-first-order and pseudo-second-order rate constants, respectively; q_e_ (mg g−1) and q_t_ (mg g−1) are the adsorption capacities at equilibrium and at a time t, respectively; h is the initial adsorption rate calculated by $h=K_{2}q_{e}$ (mg/(g min)).

### Data analysis

The processing of the FTIR spectra was performed using Omnic^®^ software. The six spectra obtained for each membrane were averaged. Baseline adjustment and noise correction were performed on each average spectrum. The corrected spectra were subsequently plotted using the software Origin Pro 8.5.

In addition, each diffraction pattern was processed and analyzed using the software Origin Pro 8.5. To verify how much the crystallinity of the BNC was being affected during the modifications, the degree of crystallinity for BNC and BNC-m was quantitatively determined using the deconvolution method (Rambo and Ferreira 2015; Ahvenainen et al., 2016). For this, the characteristic peaks on samples were found by multiple peak fit in the software and adjusting each peak to a Gaussian function, obtaining satisfactory convergences with coefficient determination greater than 0.99 in all cases.

Then, the area under the curve was found for each peak to quantitatively elucidate the crystallinity of each membrane. The crystallinity was determined using Equation 9, which establishes a relationship between the above areas (Ahvenainen et al., 2016).

$$\%Crystallinity=\frac{A_{Crystrallinepeaks}}{A_{Crystrallinepeaks}+A_{Amorphouspeak}}x100$$ (9)

The figures for the removal kinetics were also processed using the software Origin Pro 8.5.

## Results and Discussion

### Oxidation Process

With the variation in the amount of oxidizing agent (NaClO) it was sought to evaluate how the incorporation of a variable number of carboxylic groups affected the degree of removal of metal ions in solution.

**FIGURE 1:**
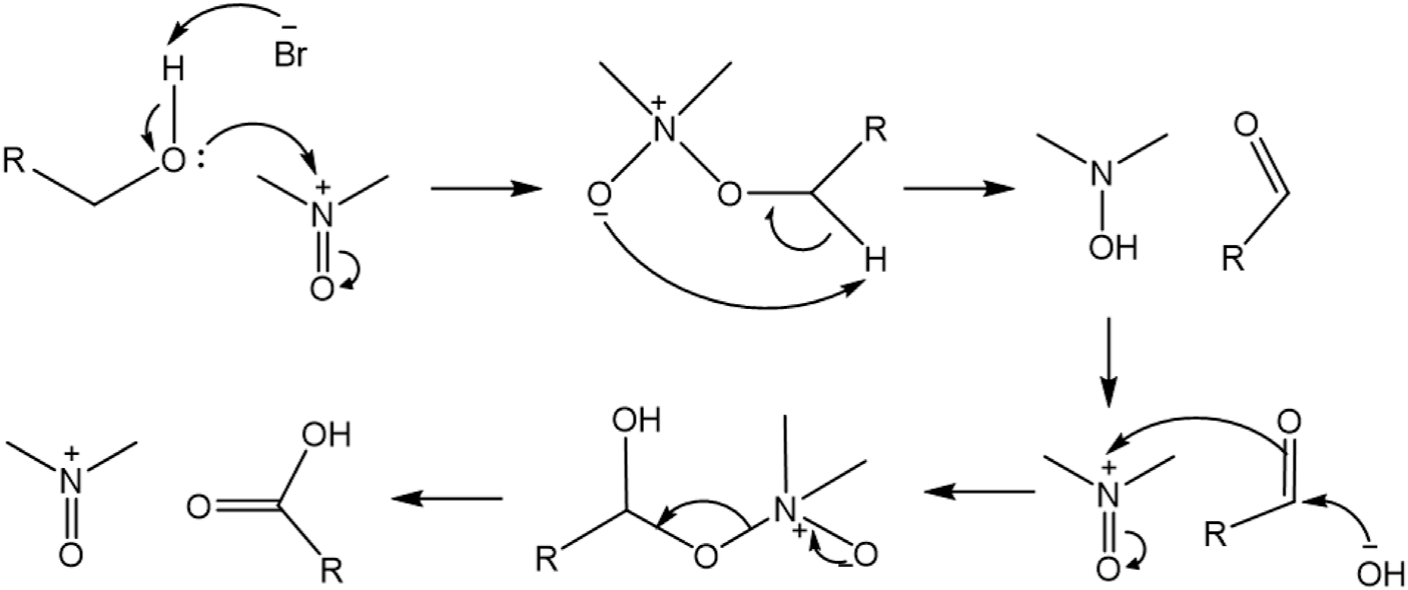
Reaction mechanism proposed for BNC oxidation.

In the oxidation process, the hydroxyl groups of the C6 primary carbons of the BNC are converted selectively to carboxylic groups as a result of the catalytic system TEMPO/NaBr/NaClO. The TEMPO is constantly in a catalytic cycle, which regenerates the reactive molecule by demand, to continue the oxidative process. Figure 1 proposes a possible reaction mechanism through which the oxidative process of the methyl hydroxyl of the BNC rings takes place. For illustrative purposes, the pyranose ring of the cellulose is replaced by R, explicitly showing only the functional groups that are involved in the oxidation process.

The mechanism proposed above shows how the lone pair of the hydroxyl group in the BNC attacks the TEMPO nucleophilically and the bromide anion eliminates the proton forming an unstable intermediate that, after an intramolecular arrangement, gives rise to the aldehyde product of the oxidation of functional group. The TEMPO enters the catalytic cycle again and returns to its oxidized form. The -OH anion generated attacks the aldehyde and then the intermediate attacks TEMPO, to finally give rise to the carboxylic acid, after a new intramolecular arrangement (Bobbitt et al., 2009)

### FTIR Spectra

**FIGURE 2:**
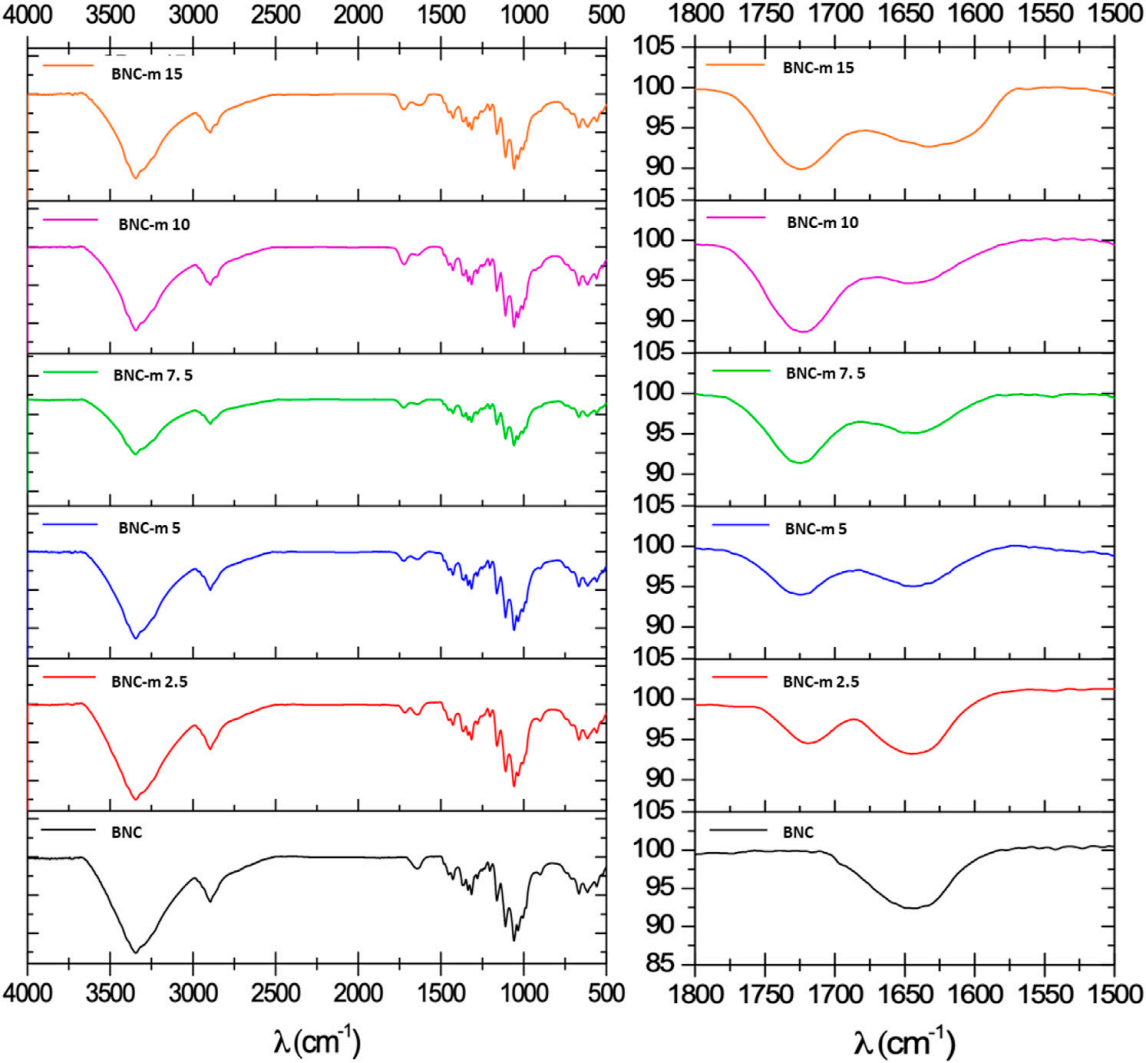
IR spectra of the BNC and BNC-m membranes. Left, full spectrum and right, extension area 1800–1500 cm^-1^.

Figure 2 shows the spectra obtained for each of membranes. The most important changes in the spectrum include the emergence of a new characteristic band at ∼1,730 cm^−1^, corresponding to the new carboxylic groups produced by the oxidative process, and the decline of the band at ∼1,650 cm^−1^, corresponding to the hydroxyl groups of the C6 carbon. This result verifies the presence of carboxylic groups produced by the oxidation of the BNC membranes when the cellulose is selectively modified in position 6.

### XRD diffractograms

**FIGURE 3:**
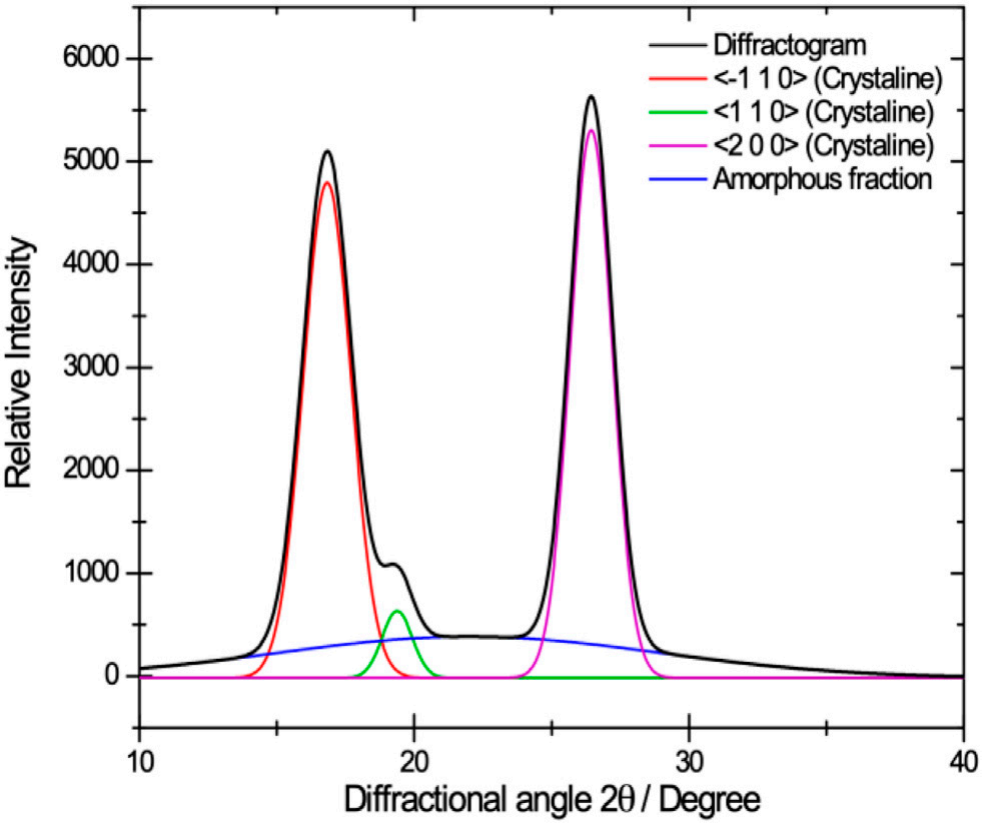
Typical diffraction pattern for BNC which illustrates the peaks corresponding to the crystalline areas and the amorphous one.

Figure 3 shows a typical diffraction pattern for cellulose. It can be observed that the diffractogram consists of four peaks, corresponding to the crystalline and amorphous areas of the polymer. The crystalline peaks correspond to the reflection on the crystalline planes < -110>, <110>, and <200> of the single cell (Cheng et al., 2009). The peak that represents the amorphous region is characterized by an amplitude that covers a large part of the diffractogram, in addition to a low intensity concerning the crystalline peaks.

**FIGURE 4:**
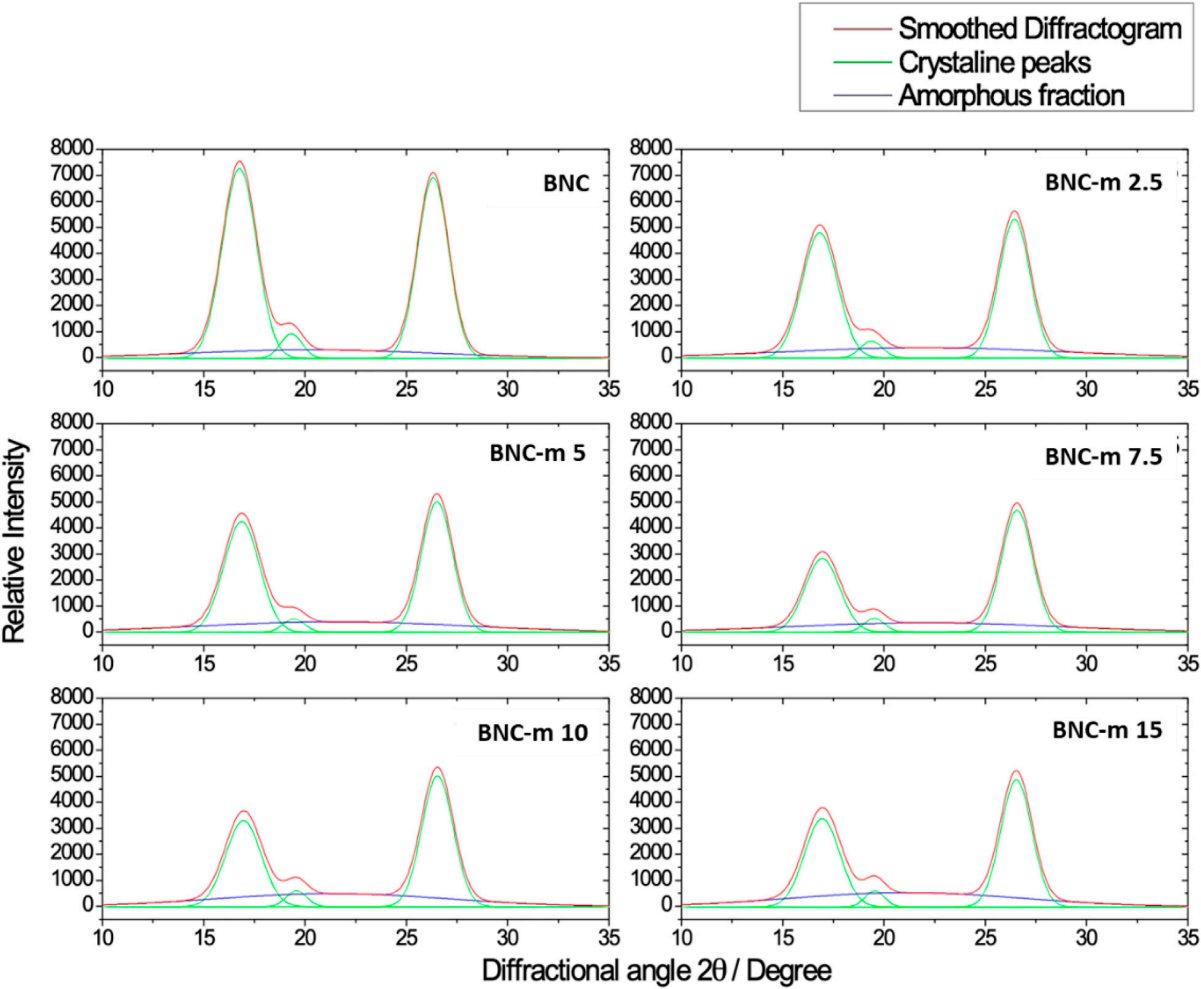
Deconvolved diffractograms for BNC and BNC-m.

Figure 4 shows the diffractograms obtained after the deconvolution for the BNC and BNC-m membranes. It can be seen that the peak corresponding to the amorphous fraction decreases as the degree of oxidation increases, however, the crystallinity was quantitatively determined to further clarify the trend.

The areas under the curve found for each peak were used to quantitatively elucidate the crystallinity of each membrane. Table 3 reports the degree of crystallinity determined for each membrane using Equation 9. It is possible to observe a marked dependence of the degree of crystallinity on the degree of modification, finding that as the degree of oxidation increases, the membrane exhibits a decrease in the degree of crystallinity, which is consistent with what is expected. At the molecular level, it is expected that as the degree of oxidation increases (which constitutes the incorporation of a greater amount of new functional groups) the packaging of the molecule is affected due to the steric impediment that these new functional groups generate. The oxidation proceeds gradually from the amorphous to the crystalline phase of cellulose and with the increase in the amount of oxidizing agent, it could be accessing the crystalline region of the cellulose. This same behavior was evidenced during the oxidation of cotton with periodate, in which the crystallinity decayed with the increase in the concentration of the oxidizing agent and with prolonged reaction time (Xu & Huang 2011).

**TABLE 3:** Degrees of crystallinity for the membranes.

Membrane	Crystallinity (%)
BNC	85.52
BNC**-m 2.5**	75.53
BNC**-m 5**	74.18
BNC**-m 7.5**	72.91
BNC**-m 10**	70.90
BNC**-m 15**	68.56

### SEM micrographs

**FIGURE 5:**
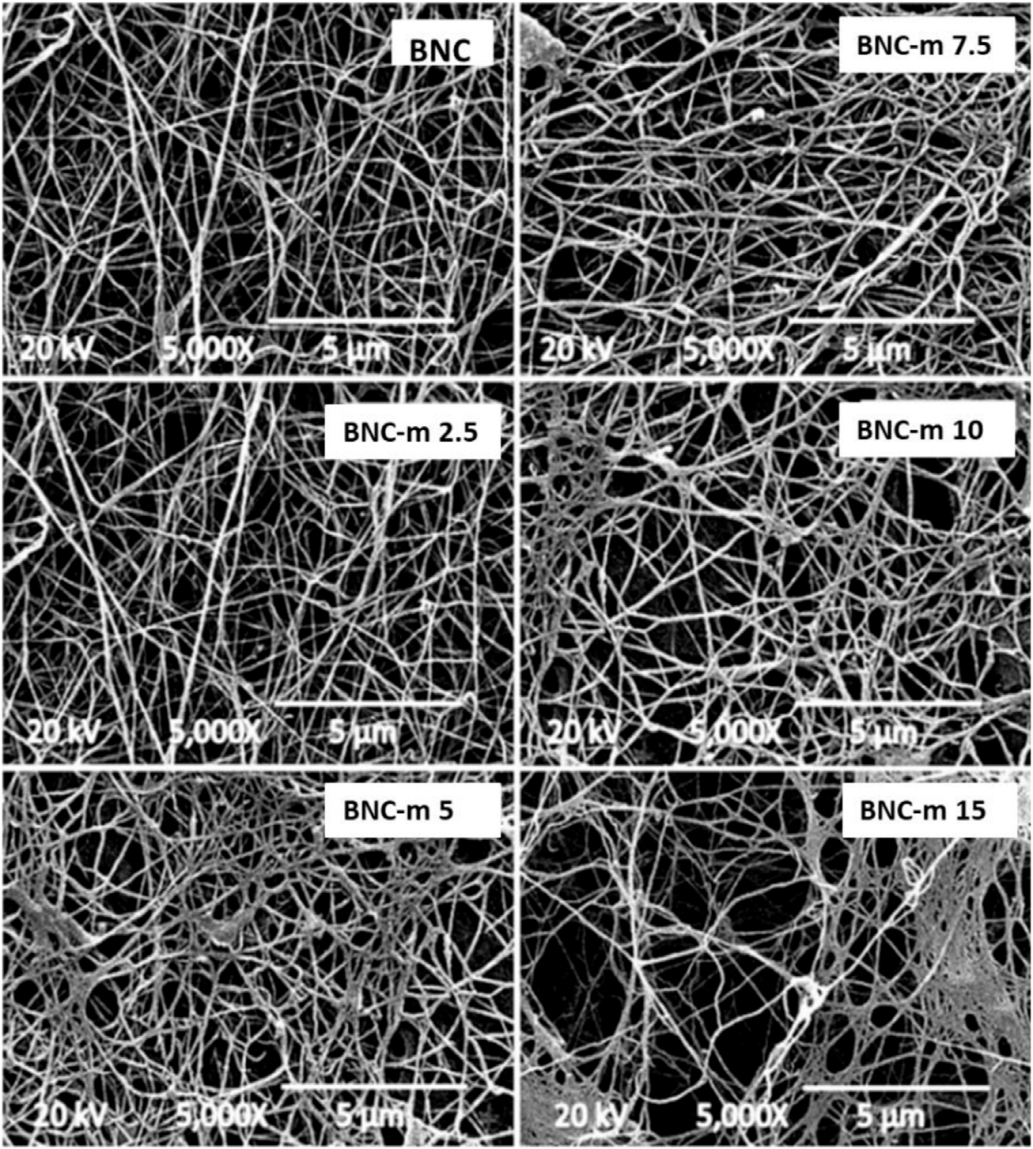
SEM micrographs at 5000X.

**FIGURE 6:**
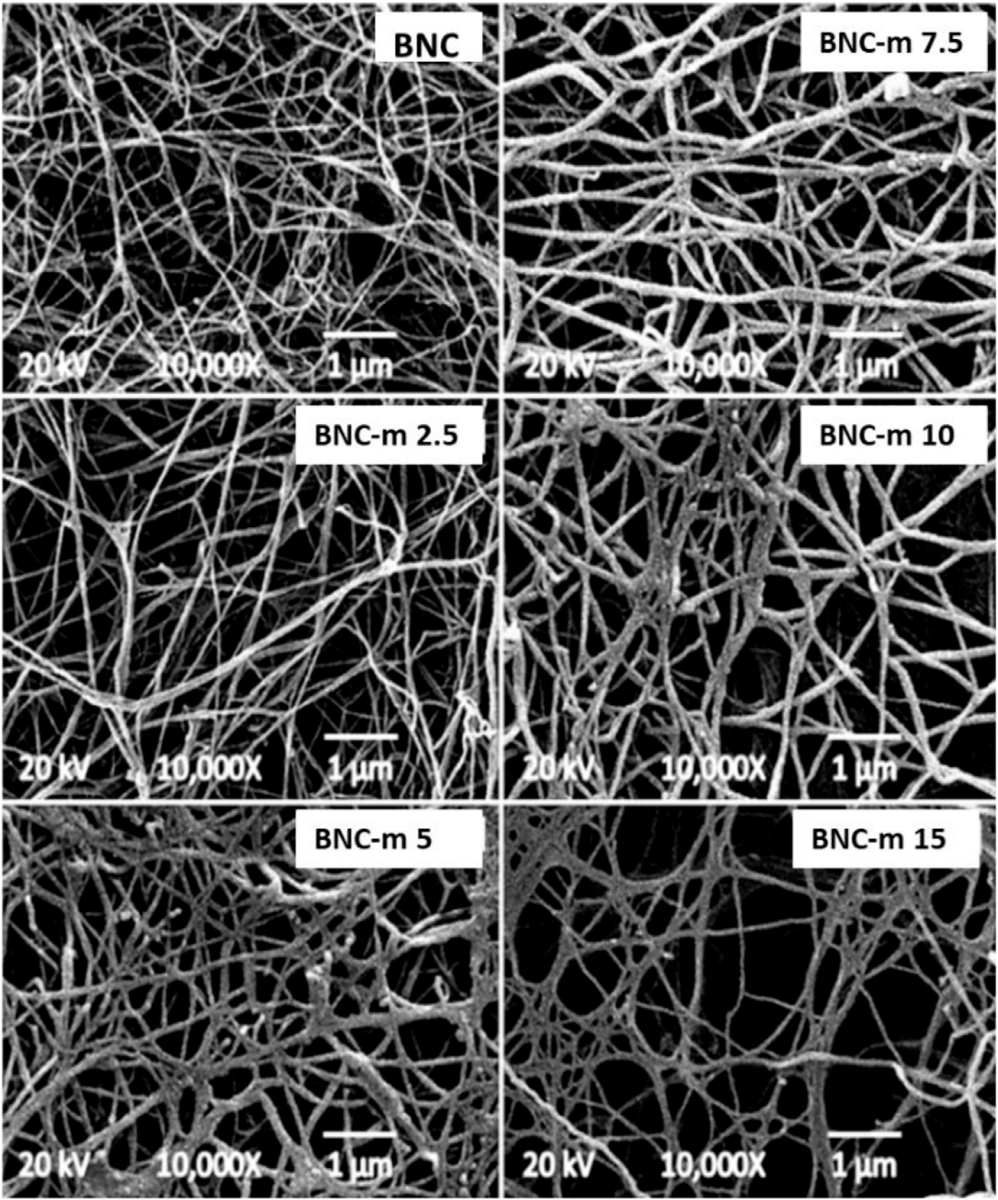
SEM micrographs at 10000X.

Figure 5 and Figure 6 present the micrographs obtained for the membranes studied, using magnifications of 5000 and 10000X, respectively. In both figures, it can be seen that as the degree of modification increases, the damage to the membranes becomes more noticeable, a situation that is more marked in the cases of the BNC-m 10 and BNC-m 15 membranes. In the specific case of the BNC-m 15, the damage caused to the network is high, with a notable interruption of the spatial organization of the fibers, as is evidenced by the appearance of “holes” in the micrograph, as a result of the depolymerization of cellulose chains due to aggressive reaction conditions to which they were subjected; this triggers the undesirable effect of loss of surface area since the active sites available for the metal removal process are reduced.

Figure 6 shows a thickening of the fibers that constitute the membranes as the degree of modification increases. The thickening of the fibers is because the incorporation of new carboxylic groups into the polymer chains increases the structural net polarity, promoting greater interaction with water molecules, which leads to a greater capacity for water retention (greater swelling of the fibers in an aqueous medium). These results are consistent with what was found by Saito and Isogai in their work (Saito and Isogai 2004).

### Mercury removal process

**FIGURE 7:**
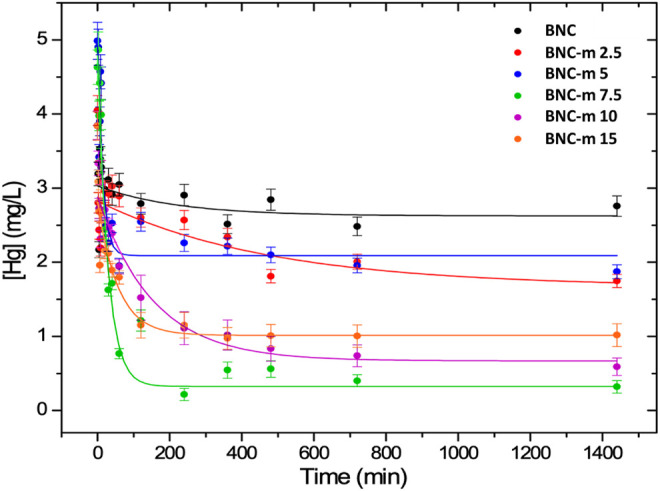
Mercury removal profiles for the BNC and BNC-m membranes.

Figure 7 shows the removal profiles for the membranes. All profiles were adjusted with good correlations to an exponential trend, represented by the continuous line. These profiles show that the modified membranes improve significantly the adsorption process of the metal ion. The presence of new carboxylic groups gives rise to interactions of greater strength (ion-ion interaction) in contrast to those that arise when there are hydroxyl groups without modification (ion-dipole interaction), a fact that promotes an improvement in the interaction between the mercury ions and the fibers, optimizing the adsorption process.

#### Kinetic study of mercury adsorption

The adsorption capacity in equilibrium qe of the BNC and BNC-m membranes and the related adsorption efficiencies in each case were calculated using Equations (3), (4). The results are shown in Table 4.

**TABLE 4:** Mercury adsorption capacity and efficiency for each membrane.

Membrane	$q_{e}$ (mg/g)	$E_{\mathrm{Ad}}$ (%)
BNC	2.50	31.61
BNC**-m 2.5**	4.69	58.79
BNC**-m 5**	5.57	58.08
BNC**-m 7.5**	8.88	92.97
BNC**-m 10**	6.83	82.59
BNC**-m 15**	5.59	73.56

It was found that the modification that results in the greatest adsorption efficiency was BNC-m 7.5 with a value of 92.97%. It is presumed that this is because, although this is not the membrane with the greatest degree of modification, it is the one that maintains its morphology concerning those with a higher degree of modification, and therefore, it could expose a greater surface area with a greater quantity of carbonyl groups for ion adsorption.

For the design and evaluation of an adsorbent, it is necessary to know its characteristic kinetic behavior. To do this, three commonly used kinetic models were applied, to investigate the adsorption mechanism and the possible parameters that control the speed with the mass transference and the chemical processes involved (Figueroa et al., 2015; Gonte and Balasubramanian 2016).

The kinetic parameters in tables 5–7 were obtained from the linearity graphs for each model used.

**TABLE 5:** Kinetic parameters for the pseudo-first-order model.

System	$q_{e}$ (mg/g)	$K_{1}$ (min^-1^)	$R^{2}$
BNC	0.9679	−0.0013	0.4220
BNC**-m 2.5**	2.2933	−0.0020	0.7326
BNC**-m 5**	1.2530	−0.0019	0.1670
BNC**-m 7.5**	4.2635	−0.0046	0.8376
BNC**-m 10**	3.6077	−0.0029	0.7694
BNC**-m 15**	1.8493	−0.0054	0.7666

**TABLE 6:** Kinetic parameters for the pseudo-second-order model.

System	$q_{e}$ (mg/g)	$K_{2}$ (g mg^-1^ min^-1^)	$R^{2}$
BNC	2.3143	0.1506	0.9861
BNC**-m 2.5**	4.4922	0.0256	0.9862
BNC**-m 5**	6.0569	0.0358	0.9968
BNC**-m 7.5**	9.0490	0.0383	0.9926
BNC**-m 10**	7.0806	0.0291	0.9978
BNC**-m 15**	5.6513	0.0876	0.9996

**TABLE 7:** Kinetic parameters for the intraparticle diffusion model.

System	$K_{\mathrm{int}1}$ (mg g^-1^min^-1/2^)	$K_{\mathrm{int}2}$ (mg g^-1^min^-1/2^)	$R_{1}^{2}$	$R_{2}^{2}$
BNC	0.0496	0.0063	0.4841	0.3075
BNC**-m 2.5**	−0.0214	0.0633	0.1119	0.5132
BNC**-m 5**	0.5174	0.0347	0.4996	0.8747
BNC**-m 7.5**	0.8985	0.0042	0.7686	0.3140
BNC**-m 10**	0.3356	0.0492	0.7124	0.8777
BNC**-m 15**	0.3416	0.0062	0.7598	0.2209

From the kinetic parameters determined, it can be concluded that the model with the best fit was the pseudo-second-order as was evidenced by the fact that the values obtained for *R* ^2^ are greater than 0.98 in all cases. In addition, the values determined for qe through the application of this mathematical model are consistent with the values reported in Table 4.

The pseudo-second-order model considers that the speed is directly proportional to the number of active sites on the surface of the adsorbent and the number of metal ions adsorbed onto the surface in time ***t*** and equilibrium (Renuka R and K 2013), which indicates that the adsorption process between the adsorbent and the mercury ions occurs through chemical interactions, which implies a process of chemisorption on the surface of the BNC and BNC-m membranes. These results also indicate that the processes that govern the interaction between the mercury ions and associated functional groups are strong. These interactions could be attributed to the adsorbent/metal ion electronic exchanges that occur in the system during the adsorption process (Pinzón and Vera 2009; Zheng et al., 2010b; Zhou et al., 2014).

#### Mercury distribution in the membranes

**FIGURE 8:**
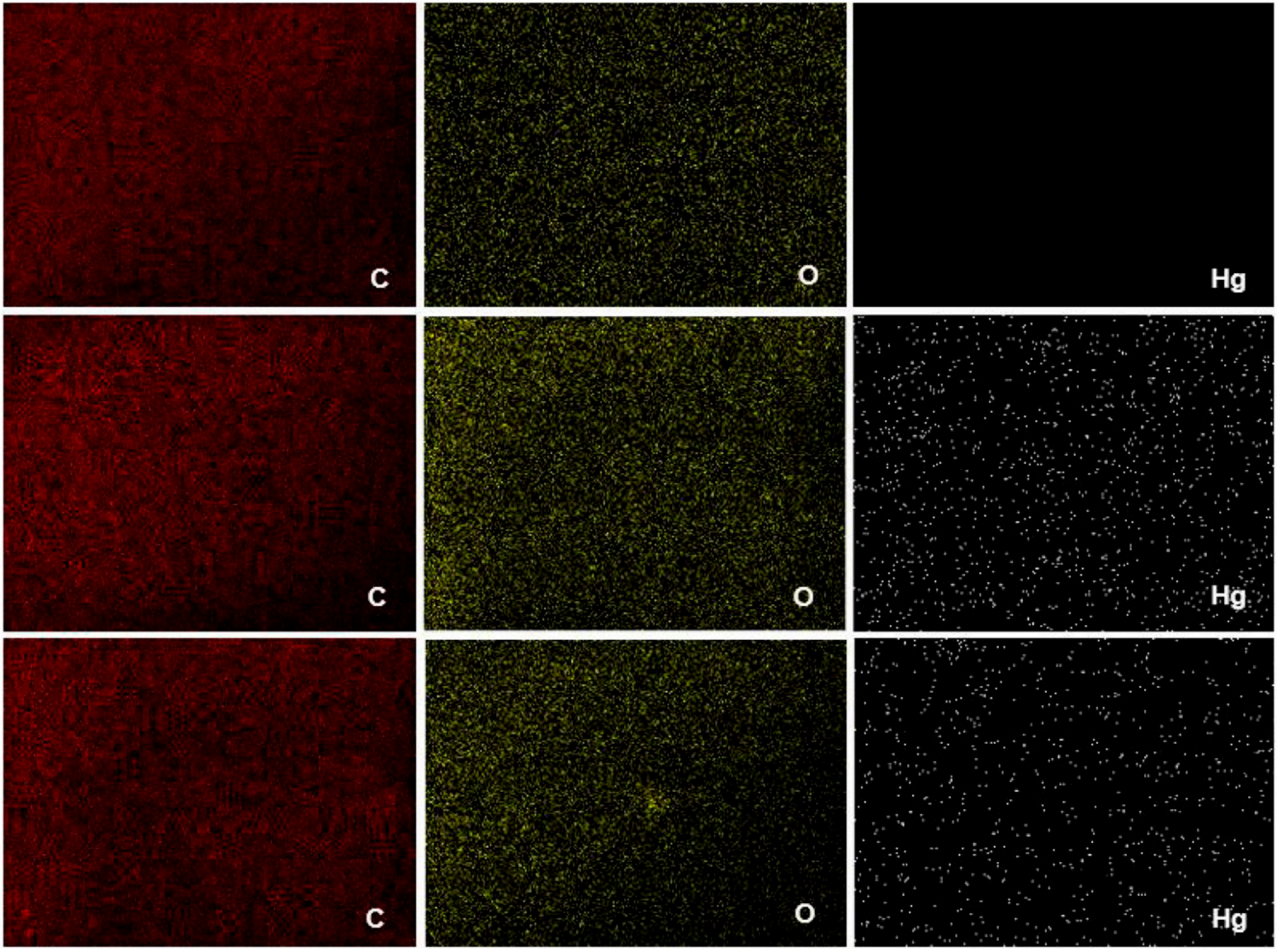
Distribution of elements by EDS mapping.

From EDS analysis, the qualitative mapping for the distribution of the elements on the membrane’s surface was found (red: Carbon (C), yellow: Oxygen (O) and white: Mercury (Hg)). This analysis was performed for the BNC, BNC-m 7.5, and BNC-m 10 membranes after mercury adsorption (Figure 8).

Figure 8 shows a homogeneous distribution of elements C and O on the surface of the membranes, which indicates that the oxidation of the membrane occurred homogeneously, in addition, a higher concentration of oxygen is evidenced on the surface of the membranes modified, which is to be expected since an additional oxygen atom is being included for each oxidized functional group. Additionally, a higher concentration of mercury on the surface is evidenced for the case of BNC-m 7.5 compared to that found for BNC-m 10, which supports the kinetically demonstrated fact that there is greater adsorption for BNC-m 7.5.

## Conclusion

In the present study, bacterial nanocellulose membranes were modified using an oxidative process mediated by TEMPO. The modified and unmodified membranes were characterized and the results of the quantitative analysis showed an improvement in mercury adsorption in the oxidized membranes, in comparison with the original membranes. The kinetic data for the mercury remotion was studied using three different kinetic models. Among these models, the pseudo-second-order described the best fit of the experimental data, indicating that the adsorption process between the adsorbent and the mercury ions occurs through chemical interactions, which implies a process of chemisorption on the surface.

In addition, it was proved that the degree of modification affects the capacity to remove mercury from the membranes since the presence of a greater number of carboxylic groups promotes greater interaction between the cellulose fibrils and the ions. However, subjecting the membranes to high concentrations of an oxidizing agent seeking a greater number of carboxylic groups affects the integrity of the fibers, which causes an undesirable effect of loss of surface area, reducing the number of active groups available for the adsorption process. This fact suggests that the modification must be carried out in such a way that it is possible to incorporate the greatest possible number of carboxylic groups, without affecting the network of fibers that conform the membranes.

The results obtained suggest that the modification of the bacterial nanocellulose membranes by oxidation transcendentally improves the mercury removal capacity, with removal percentages close to 93%, outlining the modified membranes as an excellent material for mercury removal in wastewater. These results suggest that this same system could potentially be used for the removal of potentially toxic compounds such as cations from different matrices.

## Data Availability

The original contributions presented in the study are included in the article/Supplementary Material, further inquiries can be directed to the corresponding author.
